# Incidental finding of rare hemoglobin: hemoglobin Bari in northeast Spain

**DOI:** 10.1515/almed-2023-0040

**Published:** 2023-07-04

**Authors:** Raquel Lahoz Alonso, Naiara Romero Sánchez, Ruth González Sánchez, Antonia Escobar Medina, Aurora M. López Martos, Marta Domínguez García, David Beneitez Pastor, Montserrat Prieto Grueso, Adoración Blanco Álvarez, Susana Urban Giralt, Patricia Esteve Alcalde

**Affiliations:** Servicio de Análisis Clínicos, Hospital Ernest Lluch, Zaragoza, Spain; Atención Primaria, Centro de Salud Daroca, Calle Luchente, Daroca (Zaragoza), Spain; Present Adress: Centro de Salud Calatayud Sur, Calatayud (Zaragoza), Spain; Unidad de Eritropatología, Servicio de Hematología, Hospital Universitari Vall d’Hebron, Barcelona, Spain; Técnico Superior Sanitario de Laboratorio Clínico y Biomédico, Unidad de Eritropatología, Laboratorios Clínicos Vall d’Hebron, Hospital Universitari Vall d’Hebron, Barcelona, Spain; Unidad de Genética Molecular Hematológica, Servicio de Hematología, Hospital Universitari Vall d’Hebron, Barcelona, Spain; Técnico Superior Sanitario de Laboratorio Clínico y Biomédico, Unidad de Genética Molecular Hematológica, Laboratorios Clínicos Vall d’Hebron, Hospital Universitari Vall d’Hebron, Barcelona, Spain

**Keywords:** HbA_1c_, hemoglobin Bari, HPLC

## Abstract

**Objectives:**

Cation exchange high-performance liquid chromatography (HPLC) is one of the techniques available for determining glycated hemoglobin (HbA_1c_) and also the method of choice for structural hemoglobinopathies screening. The objective of this case is to show how in a routine HbA_1c_ test it is possible to incidentally find a hemoglobinopathy.

**Case presentation:**

In a routine blood analysis, an abnormal value for the hemoglobin A2 (HbA_2_) was obtained during the study of HbA_1c_ with HPLC on the ADAMS™ A1c HA-8180T. After suspecting it could be due to the presence of a hemoglobinopathy, the study of possible variants was expanded using electrophoresis and HPLC on the Hydrasys and Variant II analysers, respectively. Since it could not be identified by these conventional methods, a genetic study was also carried out using Sanger sequencing. The patient presented a low HbA_2_ (1.3 %) and a 24.9 % variant with a retention time of 1.95 min, compatible with alpha-globin chain variant. In the genetic study, the pathogenic variant c.138C>G was detected in the *HbA*_*2*_ gene in heterozygosis, which resulted in the expression of the structural hemoglobinopathy known as hemoglobin Bari.

**Conclusions:**

The initial screening for structural hemoglobinopathies allows its identification or suspicion especially when it was performed with HbA_1c_ analysis, requiring subsequent confirmation and diagnosis by other techniques.

## Introduction

The determination of glycated hemoglobin, also known as hemoglobin A_1c_ (HbA_1c_), is recommended for the diagnosis of diabetes mellitus with a threshold of ≥6.5 %, as well as for the monitoring of this pathology [1]. This can be done by enzymatic, immunological or separation tests such as chromatography or electrophoresis.

Cation exchange high-performance liquid chromatography (HPLC) is the method of choice for structural hemoglobinopathies screening and quantifying hemoglobin A2 (HbA_2_) and fetal hemoglobin (HbF) [2].

Hemoglobin A (HbA) consists of four subunits, two alpha chains encoded by genes on chromosome 16 (hemoglobin alpha 1 (*HBA1*) and hemoglobin alpha 2 (*HBA*_*2*_)) and two beta chains encoded by a gene on chromosome 11 (hemoglobin subunit beta (*HBB*)). Each of these genes can have genetic variants. It is estimated than seven per cent of the population has a variant form of HbA. The clinical presentation is heterogeneous, ranging from hemolytic anemia and reticulocytosis to clinically silent [3, 4]. Molecular genetic testing to find the causative mutation is recommended once the suspected hemoglobinopathy has been identified [2].

Automated systems are now available that provide information on HbF, HbA_2_, HbA0, and flag abnormal peaks. The advantage of this is that it offers both the high performance of HbA_1c_ analysis and the added value of detecting the presence of hemoglobinopathies.

We present a case of a clinically healthy individual with a measured low HbA_2_ due to a rare hemoglobin variant and the confirmed genetic cause.

## Case presentation

A 40-year-old man without classic symptoms of diabetes, originally from north-eastern Spain (Zaragoza), with no medical or family history of interest, was asked to have a control blood test as a preventive measure in primary care. Whole blood samples were collected in K_2_ EDTA anticoagulant tubes (Vacutainer™ Becton-Dickinson, Rutherford, NJ, USA).

The HbA_1c_ assay was performed on the ADAMS™ A1c HA-8180T (ARKRAY, Inc., Kyoto, Japan), a high-pressure liquid chromatography system designed to separate and quantify HbA_1c_ while detecting hemoglobin variants, HbA_2_ and HbF. Before starting to work with the equipment, various internal controls are analysed to determine different levels of HbA_1c_, HbA_2_ and HbF to verify its correct operation.

**Figure 1:**
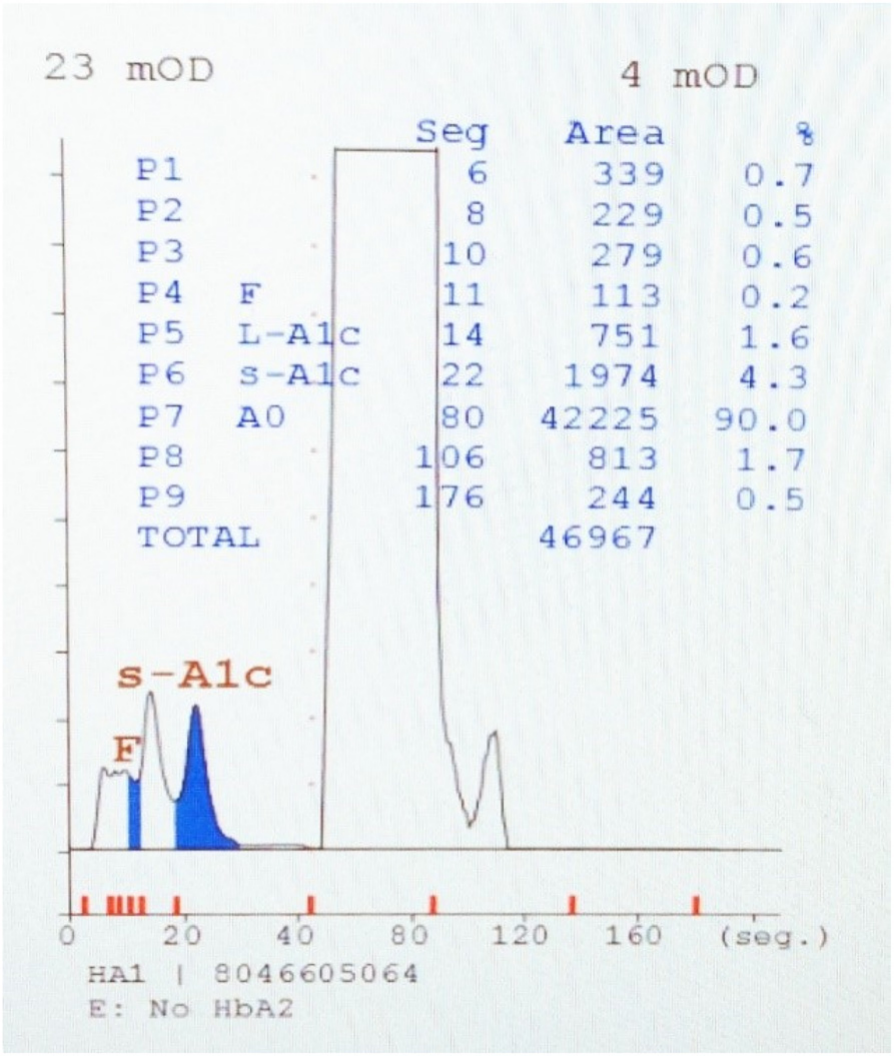
Results obtained with the Arkray ADAMS A1C HA-8180T analyser.

The patient’s level of HbA_1c_ was unremarkable (4.5 %), but the HbA_2_ level was deemed not to be reportable (Figure 1). The complete blood count results were within normal limits.

**Figure 2:**
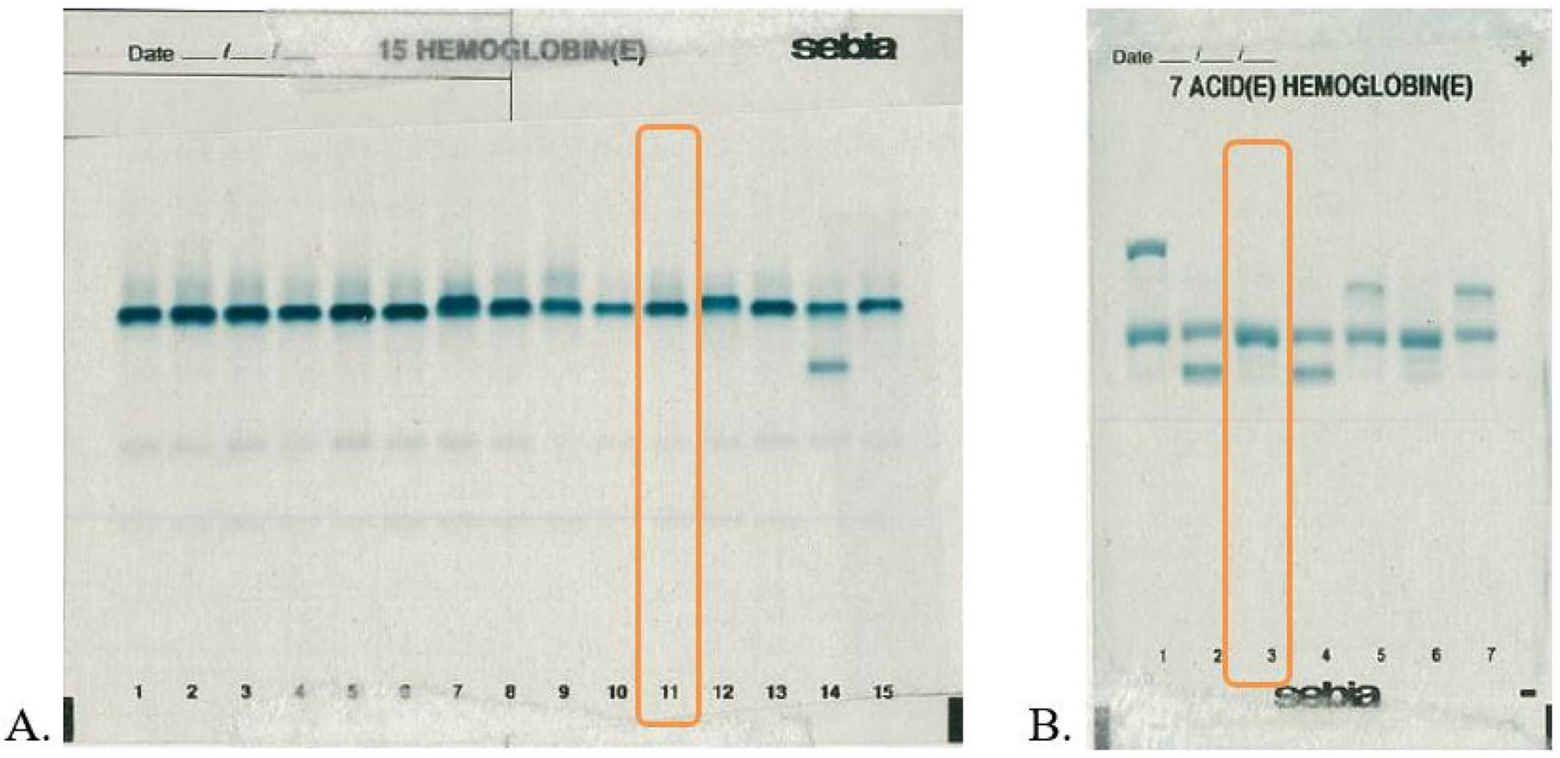
Electrophoresis study, (A) at alkaline pH (channel 11), and (B) at acid pH (channel 3) with the Hydrasis analyser.

Hemoglobin electrophoresis was therefore performed to investigate explanatory hemoglobin variants using the Hydrasys analyser (Sebia Hispania^®^). When electrophoresed at alkaline pH, the samples showed a normal Hb migration pattern. Additional testing at acid pH revealed an abnormal band that did not separate from HbA1 (Figure 2).

**Figure 3:**
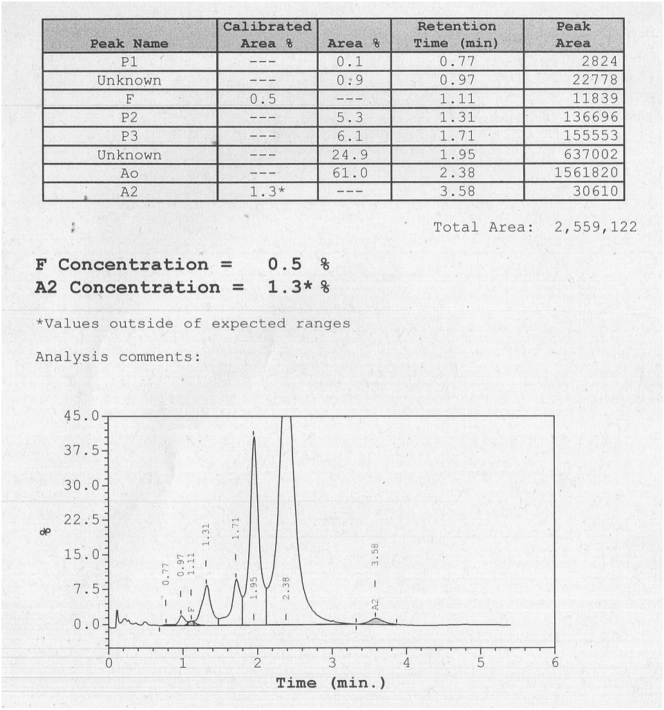
HPLC results obtained with the Variant II analyser.

After this preliminary analysis, the sample was submitted to additional testing by HPLC in the Variant II analyser (Bio-Rad^®^). The chromatogram shows a peak with a fraction of 24.9 % and retention time 1.95 min, compatible with alpha-globin chain variant (Figure 3).

Given the abnormal peak, genetic study was performed. Sanger sequencing of the *HBA1* and *HBA*_*2*_ genes was performed, studying the exonic and flanking regions of both genes. It was identified a pathogenic variant in the exon 2 of *HBA*_*2*_, the gene encoding the hemoglobin alpha 2 chain, in heterozygosis. The single nucleotide variant c.138C>G (cytosine to guanine) at coding position 46 results in a single aminoacid substitution from histidine to glutamine. This structural variant has been previously reported as hemoglobin Bari.

**Figure 4:**
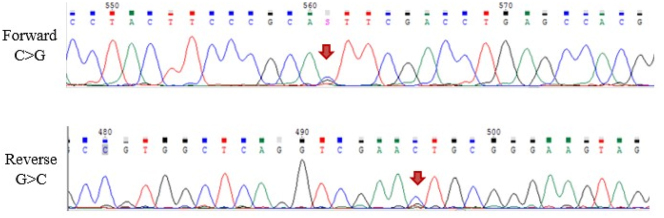
Mutation detected in the *HBA*_*2*_ gene sequencing.

The mutation in the *HBA*_*2*_ gene sequence is shown in Figure 4.

## Discussion

The suspected presence of a hemoglobinopathy in the patient during HbA_1c_ analysis led to the definitive identification of a structural variant of the alpha-globin chains, known as hemoglobin Bari. To our knowledge, this is the second reported case of this variant. The gene involved in this case was the *HbA*_*2*_ gene. Hemoglobin Bari was first discovered and described in a healthy 21-year-old man from southern Italy (Calabria) [5]. In both patients the level of the variant present was similar, 20 % in the first case and 24.9 % in the current case.

Although the aminoacid substitution that occurs in this variant implies a distal contact with the heme group, the hemoglobin molecule appeared to be virtually stable and functionally normal, which would explain the similar behavior with HbA in the HPLC study with the Variant II analyser, since it elutes a few seconds before. Furthermore, the functional parameters being studied in the sample (P_50_ O_2_, cooperativity, and Bohr effect) were similar to normal control samples [5]. These findings explain the absence of pathology in both individuals.

Marinucci M. et al. found that the electrophoretic behavior of the variant was the same as for HbA, although the abnormal alpha globin chains moved at pH 6 more anodically than normal ones [5]. This also occurs in our case, since electrophoresis at acid pH showed an abnormal band that did not separate from hemoglobin A1, as can be seen in Figure 2, which migrates more anodically.

The use of two different HPLC analysers has allowed the identification of the abnormal hemoglobin variant peak, as well as the quantification of HbA_2_. The main difference between both lies in the elution time, being 3.5 min for the ADAMS™ A1c HA-8180T and 6.5 min for the Variant II. In this way, the Variant II analyser, by processing the sample in a longer time, has made it possible to correctly separate the anomalous peak of the hemoglobin Bari variant from HbA and to quantify the low value of HbA_2_. In heterozygotes for structural α chain variants the three types of adult hemoglobins will be affected [2], so it could be that the abnormal HbA_2_ is not correctly identified and quantified, thus presenting a lower HbA_2_ level.

For each hemoglobinopathy, its prevalence varies according to the different regions of the world, although they are more common in populations from the Mediterranean and tropical areas of Africa and Asia [6]. In our case, as in the previous one [5], both individuals were from Mediterranean countries. The fact that only these two cases have been reported could be due to the absence of symptoms, so the true prevalence of this variant could be underestimated. Despite the asymptomatic nature of this pathology, its presence in patients of reproductive age is important for potential genetic counselling [2]. For this reason, it would be interesting to extend the study to the patients’ relatives, but for now these data are not available since the patient has not agreed to carry out this study in his descendants.

Hemoglobin variants can interfere with HbA_1c_ measurement, although most assays in use are not affected by the most common variants (S, C, D, and E). However, there are conflicting results for some analysers [7]. In clinical practice, the presence of a hemoglobin variant with extreme HbA_1c_ values (<4 % or >16 %) without agreement with glucose levels should be suspected [8]. In addition to the presence of less common variants that can interfere with HbA_1c_ measurement [8, 9], there are variations based on race or ethnicity and other conditions. In cases associated with increased red blood cell turnover (sickle cell anemia, pregnancy (second and third trimester), glucose-6-phosphate dehydrogenase deficiency, hemodialysis, recent blood loss, transfusion, or erythropoietin therapy), postpartum, HIV treated with certain protease inhibitors and reverse transcriptase inhibitors and iron deficiency anemia, results may be altered [10]. Even though our patient presented glucose and HbA_1c_ levels within normal limits, we believe that the HbA_1c_ may have been underestimated due to the presence of the hemoglobin Bari variant, as a value very close to 4 % was obtained. No other conditions were identified that could confound the association between HbA_1c_ levels and glycemia. Further studies are needed to confirm this phenomenon.

In conclusion, structural hemoglobinopathies screening while performing the HPLC HbA_1c_ study allows their detection, especially in asymptomatic patients with no history of interest. In these cases, it will be necessary to extend the study in order to confirm the results and make a definitive diagnosis. It must also be taken into account that their presence may interfere with the measurement of HbA_1c_, requiring further investigation if there is a discrepancy between HbA_1c_ values and glycemia.

## Learning points

1. Structural hemoglobinopathies screening while performing the HPLC HbA_1c_ study allows their detection.
2. The presence of hemoglobin variants may interfere with the measurement of HbA_1c_ by HPLC. If there is a discrepancy between HbA_1c_ and glycemia values, it requires further investigation.
3. Hemoglobin Bari is a rare structural hemoglobinopathy cause by a mutation in the *HBA*_*2*_ gene, being asymptomatic for the heterozygous carriers of the variant.
